# Evaluating the protective effectiveness and risk factors of ursodeoxycholic acid on COVID-19 among outpatients

**DOI:** 10.3389/fphar.2024.1381830

**Published:** 2024-07-31

**Authors:** Di Li, Qimei Fang, Zhiwei Chen, Jing Tang, Haoling Tang, Nan Cai, Ke Qiu, Mingyang Zhu, Xuemei Yang, Lu Yang, Yujie Yang, Yong Huang, Xiaomei Lei, Huanhuan Zhang, Qiankai Lin, Qiang Mao, Te Xu, Yan Li, Yang Zheng, Mingli Peng, Peng Hu

**Affiliations:** ^1^ Department of Infectious Diseases, Key Laboratory of Molecular Biology for Infectious Diseases (Ministry of Education), Institute for Viral Hepatitis, The Second Affiliated Hospital of Chongqing Medical University, Chongqing, China; ^2^ Department of Pharmacy, The Second Affiliated Hospital of Chongqing Medical University, Chongqing, China

**Keywords:** COVID-19, SARS-CoV-2, UDCA, outpatients, preventive efficacy, risk factors

## Abstract

**Objective:** This study aimed to assess the chemopreventive effect of ursodeoxycholic acid (UDCA) against COVID-19 and to analyze infection risk factors, symptoms, and recovery in outpatients with UDCA exposure.

**Methods:** The study enrolled outpatients prescribed UDCA from the Second Affiliated Hospital of Chongqing Medical University, China, between 01 July 2022, and 31 December 2022. Data on demographics, comorbidities, and drug combinations were collected using electronic medical records. COVID-19 infection, symptoms, severity, prognosis, vaccinations, and UDCA administration were surveyed by telephone interviews. UDCA non-users served as controls and were matched in a 1:2 ratio with UDCA users using propensity score matching with the nearest neighbor algorithm. Infection rates, symptomatology, severity, and prognosis were compared between matched and control cohorts, and risk factors and infection and recovery symptoms were analyzed in UDCA-exposed outpatients.

**Results:** UDCA-exposed outpatients (n = 778, 74.8%) and matched UDCA users (n = 95, 74.2%) showed significantly lower SARS-CoV-2 infection rates than control patients (n = 59, 92.2%) (*p* < 0.05). The matched UDCA group exhibited substantially lower fever, cough, sore throat, and fatigue rates than controls (*p* < 0.05). Participants with UDCA exposure generally experienced mild symptoms, while those without UDCA had moderate symptoms. The matched UDCA group also had significantly shorter durations of fever and cough (*p* < 0.05). Risk factors such as age over 60, less than 1 month of UDCA administration, diabetes mellitus, and coronary artery disease significantly increased SARS-CoV-2 infection rates (*p* < 0.05), while smoking led to a decrease (*p* < 0.05). Hypertension was associated with a prolonged COVID-19 recovery (*p* < 0.05), while smoking, vaccination, and fatty liver disease were associated with shorter recovery periods (*p* < 0.05). The main symptoms in the full UDCA cohort were fever, cough, and sore throat, with fatigue, cough, and hyposthenia being the most persistent.

**Conclusion:** UDCA demonstrated chemopreventive effect against SARS-CoV-2 in outpatients by significantly reducing infection incidence and mitigating COVID-19 symptoms, severity, and recovery duration. Old age, short UDCA course, and comorbidities such as diabetes mellitus and CAD increased infection rates, while hypertension prolonged recovery. Smoking, vaccination, and fatty liver disease reduced infection rates and shortened recovery. UDCA had minimal impact on symptom types. Larger and longer-term clinical studies are needed further to assess UDCA’s effectiveness in COVID-19 prevention or treatment.

## Introduction

Despite the World Health Organization (WHO) officially declaring the end of the COVID-19 pandemic, the threat posed by SARS-CoV-2 to human health continues. The virus and its new variants, including the recently identified “Pirola” or BA.2.86 variant, have resurfaced sporadically in various countries, exhibiting a high incidence trend (Contini et al., 2023; Lippi et al., 2023; Pagani et al., 2023). First detected in Denmark in late July 2023, this variant, characterized by numerous mutations that aid immune evasion, has emerged in several countries, raising concerns about a new pandemic wave (Looi, 2023; Mahase, 2023). However, vaccines may be less effective against Omicron BA.1 and BA.2 variants, especially in immunocompromised patients (John et al., 2021; John et al., 2022; John et al., 2023a; Ferreira and John, 2023). Consequently, there is an increasing demand for effective medications to prevent and mitigate severe COVID-19 cases.

Research and development of COVID-19 therapeutics have focused on creating new drugs and repurposing existing ones. However, adverse reactions limit their application in specific patient groups. For example, remdesivir, known to cause transaminase elevations, is less suitable for patients with chronic liver disease (Zampino et al., 2020). Nirmatrelvir/ritonavir, while generally low risk for hepatotoxicity, is contraindicated in patients with decompensated cirrhosis (Wong et al., 2023). Although effective against earlier variants, monoclonal antibodies face challenges in administration and cost, and their efficacy is reduced with newer variants such as BA.Q.1 (Wang et al., 2022). Thus, the urgent need for new, safe, effective, easily administered, affordable therapies must still be met.

Recent studies have highlighted angiotensin-converting enzyme 2 (ACE2) modulators and the farnesoid X receptor (FXR) as potential COVID-19 targets (Gaziano et al., 2021). Inhibition of FXR, through compounds such as z-guggulsterone and ursodeoxycholic acid (UDCA), down-regulates ACE2 in various tissues (Brevini et al., 2023). In particular, UDCA exposure has been associated with improved clinical outcomes in liver transplant recipients and patients with chronic liver disease after SARS-CoV-2 infection (Brevini et al., 2023). A larger cohort study also found that UDCA exposure is associated with reduced incidence and severity of COVID-19 in cirrhosis patients (John et al., 2023b). However, based on limited or specific cohorts, these findings require further research to establish UDCA’s chemopreventive effects against SARS-CoV-2 in a broader population. Reports on risk factors and symptoms of COVID-19 in outpatients exposed to UDCA are also scarce.

This study investigates the correlation between UDCA usage and COVID-19 treatment effects, including infection occurrence, symptomatology, disease severity, and prognosis, while identifying possible risk factors and symptoms in outpatients exposed to UDCA.

## Methods

### Study design

This retrospective cohort study was conducted at the Second Affiliated Hospital of Chongqing Medical University, China, from 01 July 2022 to 31 December 2022. The study analyzed three cohorts: the full UDCA-exposure group (patients who took daily UDCA), the matched UDCA-exposure group, and the UDCA non-exposure group (patients who did not take daily UDCA). Matching was conducted by propensity score matching (PSM). Outpatients prescribed UDCA were identified and screened. Exclusion criteria were patients who 1) refused to participate in the telephone survey, 2) lost of follow-up, 3) had death not related to COVID-19, 4) had incomplete information, and 5) documented SARS-CoV-2 infection before the initiation of UDCA administration. The UDCA capsule (250 mg) produced by Losan Pharma GmbH (Neuenburg, Germany) and the UDCA tablet (50 mg) produced by Shanghai Pukang Pharmaceutical Co. (Shanghai, China) were prescribed. The study was approved by the Institutional Review Board of The Second Affiliated Hospital of Chongqing Medical University (approval number: 97/2023) on 26 May 2023.

### Data collection

Demographic characteristics, including age, sex, body mass index (BMI), social history, diagnosis, comorbidities, and medications, were collected from the electronic medical record system. Telephone follow-up surveys were conducted according to a standard questionnaire (Supplementary Table S1), from 08 February 2023 to 12 March 2023, with subjects to collect information on COVID-19 infections, symptoms, severity, prognosis, vaccination, and the use of UDCA.

### Primary outcome

The primary outcome was SARS-CoV-2 infection, determined by positive results of the SARS-CoV-2 nucleic acid and antigen test.

### Secondary outcome

Secondary outcomes were the assessment of COVID-19 symptoms, severity, and prognosis.

### Propensity score matching

The PSM method was utilized to reduce confounding bias between groups. This involved using logistic regression with UDCA exposure as the dependent variable and various relevant covariates as independent variables to estimate propensity scores. These covariates included age, sex, BMI, smoking and alcohol habits, vaccination status, hepatobiliary diseases, and comorbidities. Matching was performed using a 1:2 nearest neighbor method, aligning each person in the UDCA non-exposure group with two in the UDCA-exposed group based on closest propensity score values.

### Statistical analysis

Continuous variables are represented as medians (interquartile range, IQR), and *p*-values were calculated using the Wilcoxon test. Categorical variables are presented as percentages (%). The *p*-values were calculated using Pearson’s chi-square (for total sample size n ≥ 40, and all expected frequencies E ≥ 5), Yates’ corrected chi-square (for n ≥ 40 with any 1 ≤ E < 5), or Fisher’s exact test (for n < 40, or E < 1). Logistic regression analysis was used to identify potential risk factors influencing SARS-CoV-2 infection in patients exposed to UDCA, and the results are presented as odds ratios (ORs) and 95% confidence intervals (CIs). The potential risk factors for COVID-19 and the recovery time in UDCA-exposed patients were determined using Cox proportional hazards regression, with the results represented as hazard ratios (HRs) and 95% CIs. Statistical analyses were conducted using SPSS (version 26), and graphs were created using GraphPad Prism (version 8.0.2) or Origin (for other symptoms and long-term symptoms radial bar graphs) (version 2021). A *p*-value of <0.05 was considered statistically significant.

## Results

### The characteristics of the patient cohorts

A total of 1,757 outpatients were prescribed UDCA. Of these, 653 individuals (37.2%) were excluded: 325 refused follow-up, 246 lost follow-up, 38 died from non-COVID-related illnesses, 28 had incomplete information, and 16 had contracted COVID-19 before taking UDCA. Among the 1,104 eligible patients, 1,040 took daily UDCA and were categorized into the full UDCA-exposure group. In the remaining 64 patients (3.6%), 46 were unwilling to take the medication, 9 forgot to pick up the medicine, 6 forgot to take the drug, and 3 had unclear reasons. They formed the UDCA non-exposure group.

Table 1 details the demographic and clinical characteristics of the full UDCA-exposure group. The median age was 56 years (IQR: 47–66 years), with women comprising 63.8%. The median BMI was 22.5 kg/m^2^ (IQR: 20.6–24.8). Smokers made up 13.8% of the group (n = 143), and alcohol consumers 8.8% (n = 91). A significant majority, 76.6% (n = 797), were vaccinated, and 52.4% (n = 545) received booster vaccinations. A total of 21.3% (n = 222) of the patients were fully vaccinated, having received two doses of an inactivated vaccine, three doses of a recombinant protein/subunit vaccine, or one dose of an adenovirus vector vaccine. UDCA was prescribed primarily for cirrhosis patients (50.6%, n = 526), and 42.7% (n = 444) had at least three liver diseases. The most common comorbidity observed was diabetes (24.4%, n = 254). The most common co-medications were antivirals (15.9%, n = 165), glucocorticoids (11.2%, n = 116), and immunosuppressants (8.8%, n = 92). The prescribed daily dose of UDCA ranged from 5 to 10 mg/kg. However, a small subset of users, 4.7% (n = 49), altered their UDCA dose, and the actual daily doses of UDCA ranged from 5 to 20 mg/kg. These patients were considered as non-adherence to UDCA.

**TABLE 1:** Baseline characteristics of UDCA-exposure group.

Characteristics		n = 1,040	Percentage (%)
Demographics	Age, median (IQR)	56.0 (47.0, 66.0)	
	<60	646	62.1
	≥60	394	37.9
	Sex		
	Female	664	63.8
	Male	376	36.2
	BMI, median (IQR)	22.5 (20.6, 24.8)	
	<18.5	73	7.0
	18.5–23.9	598	57.5
	≥24.0	369	35.5
	Smoking		
	Non-smoker	897	86.3
	Smoker	143	13.8
	Drinking		
	Non-drinker	949	91.3
	Drinker	91	8.8
	Vaccination situation		
	No vaccination	243	23.4
	Incomplete vaccination	30	2.9
	Complete vaccination	222	21.3
	Booster vaccination	545	52.4
Hepatobiliary diseases	Cirrhosis	526	50.6
	Hepatic insufficiency	376	36.2
	AIH	353	33.9
	Cholestasis	310	29.8
Hepatobiliary diseases	PBC	279	26.8
	Viral hepatitis	178	17.1
	Cholelithiasis	167	16.1
	Fatty liver	77	7.4
	DILI	73	7.0
	Jaundice	66	6.3
	Hepatoma	57	5.5
	Number of disease		
	1	273	26.3
	2	323	31.1
	>2	444	42.7
Comorbidities	Hypertension	153	14.7
	Diabetes	254	24.4
	Osteoporosis	114	11.0
	AID	97	9.3
	Hyperlipidemia	87	8.4
	Pneumonia	84	8.1
	CAD	124	11.9
	Cancer	33	3.2
	Number of comorbidity		
	0	476	45.8
	1	305	29.3
	>1	259	24.9
Drug combinations	Antiviral	165	15.9
	Glucocorticoid	116	11.2
	Immunosuppressant	92	8.8
Drug combinations	Statin	46	4.4
	Spironolactone	46	4.4
	CCB	41	3.9
	ARB	35	3.4
	Prophylactic drug^a^	35	3.4
	Number of drug		
	0	629	60.5
	1	279	26.8
	>1	132	12.7

^a^ Prophylactic drugs include Thymalfasin, Thymopentin, Human Immunoglobulin (pH4), and Huoxiangzhengqi liquid.

UDCA, ursodeoxycholic acid; BMI, body mass index; AIH, autoimmune hepatitis; PBC, primary biliary cirrhosis; DILI, drug induced liver injury; CAD, coronary artery disease; AID, autoimmune disease; CCB, calcium channel blockers; ARB, angiotensin receptor blocker.

These 64 patients with UDCA non-exposure were used as controls and matched in a 1:2 ratio with 128 patients within the full UDCA-exposure group. All demographic and clinical variables were well matched in the matched arms, as shown in Table 2.

**TABLE 2:** Baseline characteristics of the matched cohorts.

Characteristics	UDCA-exposure group (full sample)	Matched UDCA-exposure group (matched sample)	Non-UDCA exposure group	*p*-value (full vs. No exposure)	*p*-value (matched vs. No exposure)
	(n = 1,040)	(n = 128)	(n = 64)		
Demographics, n (%)					
Age, median (IQR)	56.0 (47.0, 66.0)	53 (40.0,66.0)	54.5 (38.3, 62.3)	0.268	0.813
<60	646 (62.1)	85 (66.4)	40 (62.5)	0.951	0.592
≥60	394 (37.9)	43 (33.6)	24 (37.5)		
Sex				0.221	0.918
male	376 (36.2)	55 (43.0)	28 (43.8)		
female	664 (63.8)	73 (57.0)	36 (56.3)		
BMI				0.040	0.126
<18.5	73 (7.0)	12 (9.4)	10 (15.6)		
18.5–23.9	598 (57.5)	85 (66.4)	33 (51.6)		
≥24.0	369 (35.5)	31 (24.2)	21 (32.8)		
Smoking	143 (13.8)	29 (22.7)	17 (26.6)	0.005	0.550
Drinking	91 (8.8)	13 (10.2)	6 (9.4)	0.864	0.864
Vaccination	797 (76.6)	49 (38.3)	18 (28.1)	<0.001	0.164
Hepatobiliary diseases, n (%)					
Cirrhosis	526 (50.6)	29 (22.7)	10 (15.63)	<0.001	0.254
Hepatic insufficiency	376 (36.2)	44 (34.4)	18 (28.13)	0.193	0.383
AIH	353 (33.9)	17 (13.3)	7 (10.94)	<0.001	0.643
Cholestasis	310 (29.8)	47 (36.7)	25 (39.06)	0.118	0.752
PBC	279 (26.8)	3 (2.3)	1 (1.56)	<0.001	1.000
Viral hepatitis	178 (17.1)	26 (20.3)	10 (15.63)	0.758	0.433
Cholelithiasis	167 (16.1)	20 (15.6)	12 (18.75)	0.571	0.584
Fatty liver	77 (7.4)	7 (5.5)	4 (6.25)	0.923	1.000
DILI	73 (7.0)	12 (9.4)	7 (10.94)	0.241	0.733
Jaundice	66 (6.3)	5 (3.9)	2 (3.13)	0.440	1.000
Hepatoma	57 (5.5)	4 (3.1)	2 (3.13)	0.598	1.000
Comorbidities, n (%)					
Hypertension	153 (14.7)	9 (7.0)	4 (6.25)	0.090	1.000
Diabetes	254 (24.4)	2 (1.6)	2 (3.13)	<0.001	0.858
Osteoporosis	114 (11.0)	6 (4.7)	3 (4.69)	0.170	1.000
AID	97 (9.3)	7 (5.5)	2 (3.13)	0.144	0.717
Hyperlipidemia	87 (8.4)	14 (10.9)	6 (9.38)	0.778	0.933
Pneumonia	84 (8.1)	0 (0.0)	0 (0.00)	0.012	
CAD	124 (11.9)	6 (4.7)	3 (4.69)	0.119	1.000
Cancer	33 (3.2)	2 (1.6)	1 (1.56)	0.726	1.000

Values are median (IQR) or number (percentage). *P* values were calculated by the chi-square test, Yates’s correction for continuity or Fisher’s exact test. Bold values signifies *p* < 0.05. UDCA, ursodeoxycholic acid; BMI, body mass index; AIH, autoimmune hepatitis; PBC, primary biliary cirrhosis; DILI, drug induced liver injury; CAD, coronary artery disease; AID, autoimmune disease; CCB, calcium channel blockers; ARB, angiotensin receptor blocker.

### Influence of UDCA exposure on the SARS-CoV-2 infection rate

**FIGURE 1:**
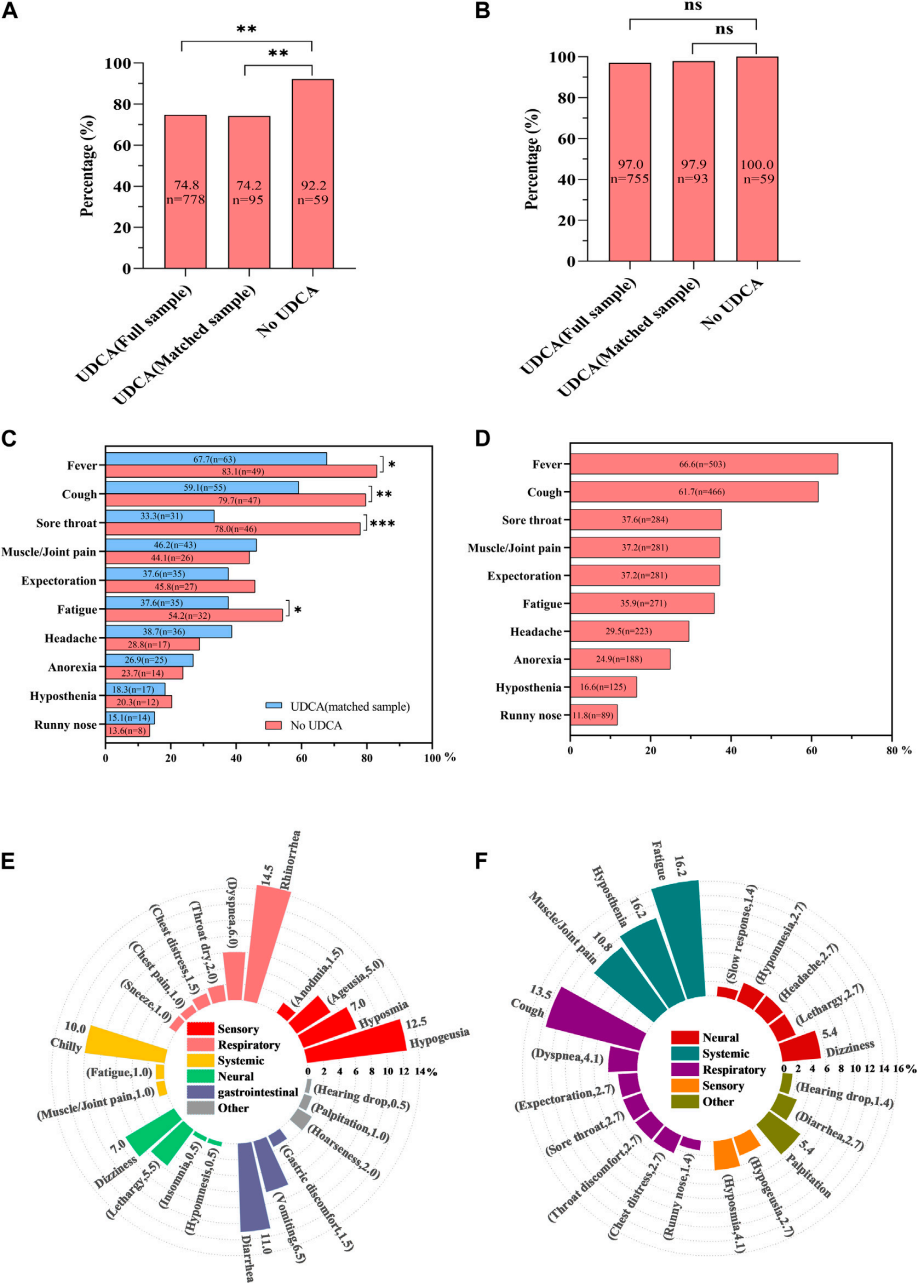
Comparative analysis of SARS-CoV-2 infection and COVID-19 symptoms in UDCA-exposed and non-exposed cohorts. **(A)** Infection rate comparison: This panel shows the infection rates of severe acute respiratory syndrome coronavirus-2 (SARS-CoV-2) across three groups: the full UDCA-exposure cohort, the matched UDCA-exposure cohort, and the UDCA non-exposure cohort. **(B)** Overall symptom rate in COVID-19: This graph illustrates the total symptom rate of novel coronavirus disease (COVID-19) in infected patients. **(C)** Main symptom proportions in matched cohorts: This section depicts the proportions of main COVID-19-induced symptoms in both matched UDCA-exposure and UDCA non-exposure cohorts. **(D)** Main symptoms in full UDCA-exposure cohort: This panel details the main symptoms of COVID-19 observed in the full UDCA-exposure group. **(E)** Other symptoms in full UDCA-exposure cohort: This graph outlines additional, less common symptoms of COVID-19 found in the full UDCA-exposure group. **(F)** Persistent symptoms in full UDCA-exposure cohort: This part of the figure shows the persistent symptoms of COVID-19 experienced by participants in the full UDCA-exposure cohort. Statistical significance: A *p*-value of less than 0.05 was considered statistically significant. * denotes *p* < 0.05, ** indicates *p* < 0.01, *** signifies *p* < 0.001, and ns represents non-significant results (*p* > 0.05).

In the full UDCA-exposure group (n = 1,040), 74.8% (n = 778) contracted SARS-CoV-2 during the study period. In the UDCA-exposure matched cohort (n = 128), the infection rate was 74.2% (n = 95). In contrast, in the UDCA non-exposure group, the infection rate was significantly higher at 92.2% (59/64). This rate was significantly higher than both the full UDCA-exposure group (92.2% vs. 74.8%, *p* = 0.002) and the matched UDCA-exposure group (92.2% vs. 74.2%, *p* = 0.003), as illustrated in Figure 1A.

### Impact of UDCA exposure on COVID-19 symptoms

In the full and matched UDCA-exposure groups, COVID-19 symptoms were observed in 97.0% (n = 755) and 97.9% (n = 93) of the patients, respectively. This compares to a 100.0% symptom occurrence (n = 59) in the UDCA non-exposure group (Figure 1B).

When analyzing specific COVID-19 symptoms, the matched UDCA-exposure group exhibited significantly lower instances of fever (67.7% vs. 83.1%, *p* = 0.037), cough (59.1% vs. 79.7%, *p* = 0.009), sore throat (33.3% vs. 78.0%, *p* < 0.001), and fatigue (37.6% vs. 54.2%, *p* = 0.045) compared to the UDCA non-exposure group (Figure 1C).

In the full UDCA-exposure cohort (n = 1,040), the ten most common symptoms were fever (66.6%, n = 503), cough (61.7%, n = 466), sore throat (37.6%, n = 284), muscle or joint pain (37.2%, n = 281), expectoration (37.2%, n = 281), fatigue (35.9%, n = 271), headache (29.5%, n = 223), anorexia (24.9%, n = 188), hyposthenia (16.6%, n = 125), and runny nose (11.8%, n = 89) (Figure 1D).

Additional symptoms affected respiratory, sensory, gastrointestinal, skin-muscular, and neural systems, including rhinorrhea, hypogeusia, diarrhea, and chills (Figure 1E). In particular, 74 participants reported experiencing at least 20 persistent COVID-19 symptoms over an average duration of 65.0 days (range: 60.0–74.0), with the most common being fatigue, cough, hyposthenia, muscle/joint pain, and dizziness, in that order (Figure 1F).

### Relationship between UDCA exposure and COVID-19 severity

**FIGURE 2:**
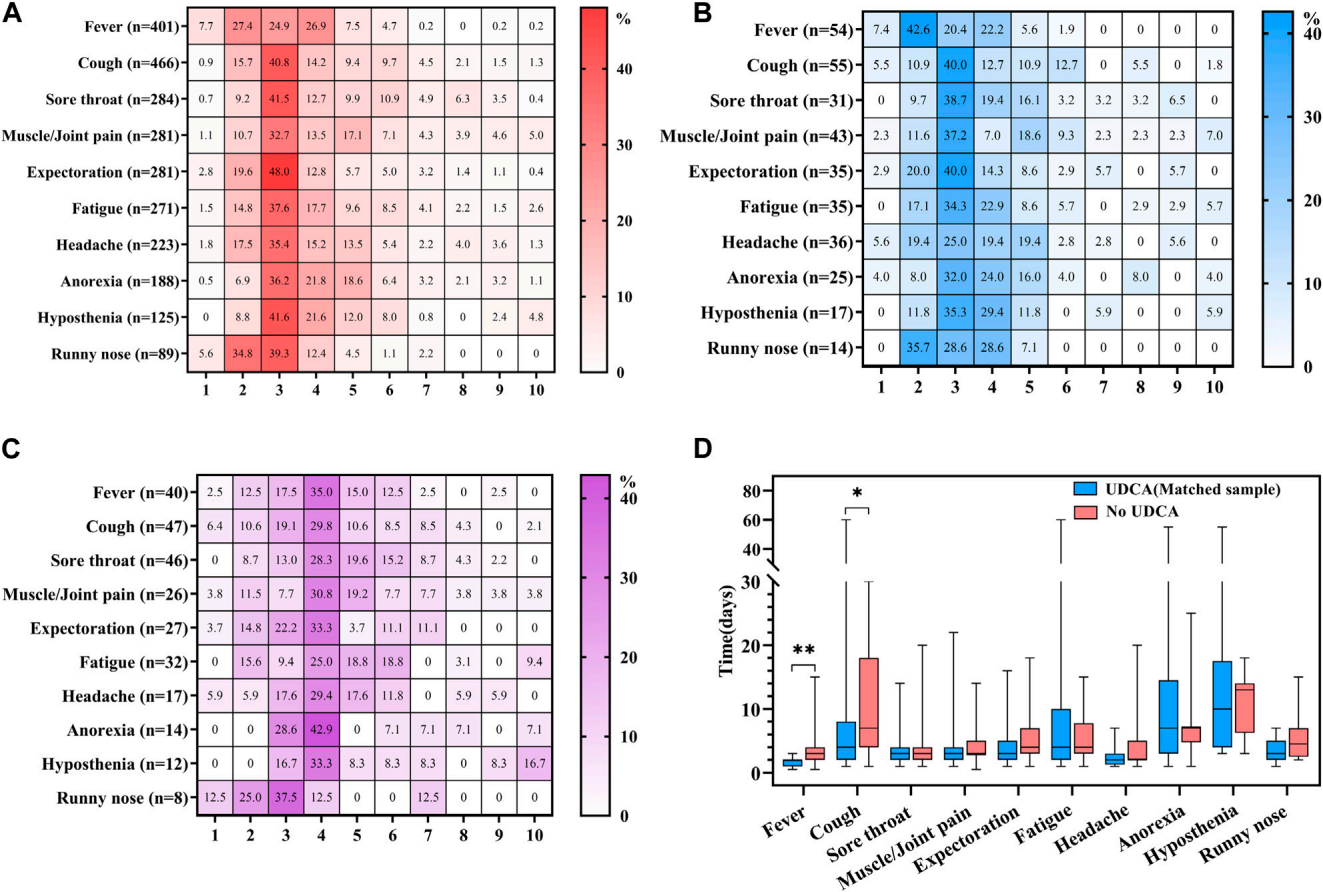
Analysis of COVID-19 symptom severity and duration in UDCA-exposed and non-exposed groups. **(A)** Severity in full UDCA-exposure group: This panel illustrates the severity of main symptoms induced by novel coronavirus disease (COVID-19) in the full UDCA-exposure group. **(B)** Severity in matched UDCA-exposure group: This section depicts the severity of main COVID-19 symptoms in the matched UDCA-exposure group. **(C)** Severity in UDCA non-exposure group: This graph shows the severity of main COVID-19 symptoms in the UDCA non-exposure group. **(D)** Duration of symptoms in matched groups: This part of the figure compares the duration of main COVID-19 symptoms between the matched UDCA-exposure and UDCA non-exposure groups. Statistical significance: A *p*-value of less than 0.05 was considered statistically significant. In the figure, * denotes *p* < 0.05 and ** indicates *p* < 0.01.

The severity of COVID-19-induced symptoms was assessed using a scale of 0–10. The patients were classified as asymptomatic with a severity rating of 0, experiencing mild symptoms with ratings of 1–3, moderate symptoms with ratings of 4–6, and severe symptoms with a rating of 7 or higher. Most participants with UDCA exposure reported mild symptoms, with severity scores ranging from 2 to 3 out of 10, in both the full and the matched UDCA-exposure groups. Common symptoms in this category included expectoration, hyposthenia, sore throat, and cough (Figures 2A, B). In contrast, individuals in the UDCA non-exposure group tended to experience more moderate symptoms, with severity scores between 4 and 5. These included anorexia, fever, cough, muscle or joint pain, and hyposthenia (Figure 2C).

### Correlation between UDCA exposure and the duration of COVID-19 symptoms

The duration of symptoms was calculated from the first report of a symptom until the return to a state of no symptoms. Among the top ten most frequent symptoms, the matched UDCA-exposure group exhibited a significantly shorter duration of fever (*p* = 0.003) and cough (*p* = 0.011) compared to the UDCA non-exposure group. Although there were no significant differences in the duration of other symptoms between the two groups, most symptoms tended to have a prolonged recovery period in the UDCA non-exposure group (Figure 2D).

### Risk factors for SARS-CoV-2 infection in patients with UDCA exposure

In analyzing the risk factors for SARS-CoV-2 infection among UDCA-exposure individuals, different trends emerged based on age, smoking habits, duration of UDCA use, and other comorbidities. In the population over 60 years old, univariate analysis did not reveal significant differences in COVID-19 prevalence compared to those under 60 years of age (OR 1.34, 95% CI 1.00–1.80, *p* = 0.051). However, a significant increase of 45% was observed in infection risk in multivariate analysis (OR 1.45, 95% CI 1.02–2.04, *p* = 0.037).

Interestingly, smoking was associated with a 38.0% reduction in infection risk in univariate analysis (OR 0.62, 95% CI 0.43–0.91, *p* = 0.014), but this association was not significant in multivariate analysis (OR 0.75, 95% CI 0.44–1.27, *p* = 0.288). Patients who received UDCA for less than 1 month had a 3.92-fold increase in infection risk in univariate analysis (OR 3.92, 95% CI 2.62–5.86, *p* < 0.001), and a 4.51-fold increase in multivariate analysis (OR 4.51, 95% CI 5.45–6.91, *p* < 0.001) compared to those who received UDCA for more than 1 month.

Our findings indicated a weak association between the etiologies of liver disease and SARS-CoV-2 infection. However, specific comorbidities, such as diabetes mellitus and coronary artery disease (CAD), were significantly associated with an increased risk of viral infection in both univariate (diabetes: OR 1.83, 95% CI 1.28–2.63, *p* = 0.001; CAD: OR 2.68, 95% CI 1.53–4.49, *p* = 0.001) and multivariate analysis (diabetes: OR 2.28, 95% CI 1.24–4.19, *p* = 0.008; CAD: OR 3.65, 95% CI 1.74–7.63, *p* = 0.001). Additionally, factors such as adherence to UDCA, vaccination status, and drug combination were not significantly associated with SARS-CoV-2 infection. Details are shown in Table 3.

**TABLE 3:** Analysis of risk factors for severe acute respiratory syndrome coronavirus-2 (SARS-CoV-2) infection with ursodeoxycholic acid (UDCA) exposure (n = 1,040).

Characteristics	Non-infection	Infection	Univariate analysis			Multivariate analysis		
	(n = 262)	(n = 778)	OR	95%CI	*p*-value	OR	95%CI	*p*-value
Demographics, n (%)								
Age, median (IQR)	55.0 (46.0, 65.0)	56.0 (48.0, 66.3)			0.327			
Age (≥60)	86 (32.8)	308 (39.6)	1.34	1.00–1.80	0.051	1.45	1.02–2.04	0.037
Sex (female)	155 (59.2)	509 (65.4)	1.31	0.98–1.74	0.068	1.23	0.82–1.85	0.326
BMI					0.732			0.653
<18.5 (vs. 18.5–23.9)	17 (6.5)	56 (7.2)	1.07	0.61–1.91	0.808	1.04	0.55–1.94	0.913
≥24.0 (vs. 18.5–23.9)	98 (37.4)	271 (34.8)	0.90	0.67–1.21	0.493	0.86	0.62–1.19	0.357
Smoking	48 (18.3)	95 (12.2)	0.62	0.43–0.91	0.014	0.75	0.44–1.27	0.288
Drinking	28 (10.7)	63 (8.1)	0.74	0.47–1.18	0.201	0.99	0.54–1.81	0.975
Vaccination situation (vs. 0)					0.345			0.188
Incomplete vaccination	10 (3.8)	20 (2.6)	0.73	0.33–1.64	0.447	0.85	0.36–2.03	0.714
Complete vaccination	47 (17.9)	175 (22.5)	1.36	0.89–2.09	0.161	1.59	0.98–2.56	0.058
Booster vaccination	140 (53.4)	405 (52.1)	1.06	0.75–1.49	0.754	1.09	0.73–1.62	0.678
UDCA status, n (%)								
Compliance (UDCA dosage)	248 (94.7)	743 (95.5)	1.20	0.61–2.22	0.577	1.08	0.45–2.18	0.823
UDCA course (<1 month)	31 (11.8)	268 (34.4)	3.92	2.62–5.86	<0.001	4.51	5.45–6.92	<0.001
Hepatobiliary diseases, n (%)								
Cirrhosis	131 (50.0)	395 (50.8)	1.03	0.78–1.37	0.829	0.93	0.57–1.52	0.779
Hepatic insufficiency	94 (35.9)	282 (36.2)	1.02	0.76–1.36	0.914	1.07	0.68–1.66	0.775
AIH	79 (30.2)	274 (35.2)	1.26	0.93–1.70	0.135	1.20	0.76–1.90	0.442
Cholestasis	88 (33.6)	222 (28.5)	0.79	0.59–1.07	0.122	0.69	0.45–1.05	0.084
PBC	64 (24.4)	215 (27.6)	1.181	0.86–1.63	0.311	1.13	0.67–1.91	0.65
Viral hepatitis	39 (14.9)	139 (17.9)	1.24	0.85–1.83	0.269	1.28	0.59–2.78	0.541
Cholelithiasis	39 (14.9)	128 (16.5)	1.13	0.76–1.66	0.550	1.12	0.67–1.86	0.674
Fatty liver	20 (7.6)	57 (7.3)	0.96	0.56–1.63	0.870	0.86	0.45–1.66	0.661
DILI	16 (6.1)	57 (7.3)	1.22	0.69–2.16	0.504	1.22	0.63–2.35	0.561
Jaundice	19 (7.3)	47 (6.0)	0.82	0.47–1.43	0.487	0.73	0.37–1.44	0.361
Hepatoma	14 (5.3)	43 (5.5)	1.04	0.56–1.93	0.910	1.00	0.46–2.15	0.998
Number of disease					0.122			0.323
2 (vs. 1)	93 (35.5)	230 (29.6)	0.85	0.59–1.23	0.390	0.87	0.51–1.48	0.605
>2 (vs. 1)	99 (37.8)	345 (44.3)	1.20	0.85–1.71	0.306	1.25	0.51–3.07	0.623
Comorbidities, n (%)								
Hypertension	47 (17.9)	106 (13.6)	0.72	0.50–1.05	0.089	0.71	0.37–1.39	0.323
Diabetes	44 (16.8)	210 (26.0)	1.83	1.28–2.63	0.001	2.28	1.24–4.19	0.008
Osteoporosis	34 (13.0)	80 (10.3)	0.77	0.50–1.18	0.228	0.90	0.46–1.77	0.753
AID	26 (9.9)	71 (9.1)	0.91	0.57–1.46	0.701	1.23	0.59–2.56	0.583
Hyperlipidemia	20 (7.6)	67 (8.6)	1.14	0.68–1.92	0.621	1.35	0.62–2.93	0.453
Pneumonia	22 (8.4)	62 (7.0)	0.95	0.57–1.57	0.826	1.16	0.57–2.37	0.685
CAD	15 (5.7)	109 (14.0)	2.68	1.53–4.49	0.001	3.65	1.74–7.63	0.001
Cancer	13 (5.0)	20 (2.6)	0.51	0.25–1.03	0.061	0.54	0.22–1.34	0.181
Number of comorbidity					0.093			0.570
1 (vs. 0)	64 (24.4)	241 (30.0)	1.46	1.04–2.05	0.029	0.97	0.54–1.76	0.931
>1 (vs. 0)	65 (24.8)	194 (24.9)	1.16	0.82–1.63	0.406	0.66	0.20–2.12	0.481
Drug combinations, n (%)								
Antiviral	37 (14.1)	128 (16.5)	1.20	0.81–1.78	0.372	1.71	0.59–4.91	0.322
Glucocorticoid	27 (10.3)	89 (11.4)	1.12	0.71–1.77	0.614	1.38	0.54–3.57	0.504
Immunosuppressant	24 (9.2)	68 (8.7)	0.95	0.58–1.55	0.836	1.19	0.46–3.05	0.722
Statin	12 (4.6)	34 (4.4)	0.95	0.49–1.87	0.886	1.17	0.40–3.43	0.779
Spironolactone	13 (5.0)	33 (4.2)	0.85	0.44–1.64	0.624	0.95	0.33–2.76	0.925
CCB	15 (5.7)	26 (3.3)	0.57	0.30–1.09	0.090	0.63	0.21–1.88	0.408
ARB	11 (4.2)	24 (3.1)	0.73	0.35–1.50	0.389	1.00	0.32–3.08	0.999
Prophylactic drug	12 (4.6)	23 (2.0)	0.64	0.31–1.29	0.211	0.74	0.25–2.18	0.583
Number of drug					0.837		-	0.877
1 (vs. 0)	70 (26.7)	209 (26.9)	0.985	0.71–1.36	0.926	0.81	0.35–1.89	0.627
>1 (vs. 0)	36 (13.7)	96 (12.3)	0.879	0.58–1.34	0.552	0.64	0.12–3.54	0.613

Values are median (IQR) or number (percentage). OR, odds ratio; CI, Confidence Interval. *P* values were calculated by logistic regression analysis. Bold values signifies *p* < 0.05. UDCA, ursodeoxycholic acid; BMI, body mass index; AIH, autoimmune hepatitis; PBC, primary biliary cirrhosis; DILI, drug induced liver injury; CAD, coronary artery disease; AID, autoimmune disease; CCB, calcium channel blockers; ARB, angiotensin receptor blocker.

### Risk factors influencing COVID-19 duration in patients with UDCA exposure

The duration of COVID-19 was calculated from the date of the first positive nucleic acid or antigen test result to the date of the first negative result. Among the 213 participants confirmed to have seroconverted through test results and exposed to UDCA, various factors influenced the duration of their illness. In the smoker population, a reduction in the duration of COVID-19 was observed, with a 63.0% reduction in univariate analysis (HR 1.63, 95% CI 1.06–2.51, *p* = 0.026) and a 97.0% reduction in multivariate analysis (HR 1.97, 95% CI 1.10–3.53, *p* = 0.023). Participants who had complete vaccinations showed a 69.0% reduction in the duration of clinical symptoms (HR 1.69, 95% CI 1.10–2.58, *p* = 0.017), and those with booster vaccinations showed a 56.0% reduction (HR 1.56, 95% CI 1.11–2.19, *p* = 0.010). The specific information of vaccinations was shown in Supplementary Table S2. Additionally, fatty liver was associated with a shortened recovery period (HR 1.89, 95% CI 1.07–3.33, *p* = 0.028). In contrast, patients with comorbidities such as hypertension experienced a significant extension in their recovery period (HR 0.61, 95% CI 0.42–0.89, *p* = 0.010). Details are shown in Table 4.

**TABLE 4:** Analysis of risk factors associated with the duration of COVID-19 and UDCA exposure.

Characteristics	Infection	Univariate analysis		Multiariate analysis	
	(n = 213)	HR (95%Cl)	*p*-value	HR (95%Cl)	*p*-value
Demographics, n (%)					
Age (≥60)	80 (37.6)	0.89 (0.66–1.16)	0.362	1.11 (0.79–1.57)	0.539
Sex (female)	142 (66.7)	0.89 (0.67–1.19)	0.436	1.15 (0.79–1.69)	0.464
BMI			0.447		0.993
<18.5 (vs. 18.5–23.9)	18 (8.5)	0.93 (0.57–1.52)	0.767	1.03 (0.59–1.81)	0.905
≥24.0 (vs. 18.5–23.9)	71 (33.3)	1.19 (0.88–1.59)	0.255	0.99 (0.69–1.43)	0.968
Smoking	24 (11.3)	1.63 (1.06–2.51)	0.026	1.97 (1.10–3.53)	0.023
Drinking	20 (9.4)	1.55 (0.97–2.48)	0.065	1.03 (0.53–1.99)	0.932
Vaccination situation			0.045		0.287
Incomplete vaccination	5 (2.3)	1.79 (0.71–4.53)	0.220	1.53 (0.45–5.25)	0.499
Complete vaccination	40 (18.8)	1.69 (1.10–2.58)	0.017	1.70 (0.98–2.96)	0.060
Booster vaccination	119 (55.9)	1.56 (1.11–2.19)	0.010	1.45 (0.92–2.28)	0.110
UDCA status, n (%)					
Compliance (UDCA dosage)	192 (90.1)	0.99 (0.79–1.24)	0.946	0.96 (0.56–1.63)	0.867
UDCA course (<1 month)	77 (36.2)	1.17 (0.89–1.55)	0.280	1.10 (0.79–1.52)	0.582
Hepatobiliary diseases, n (%)					
Cirrhosis	94 (44.1)	1.00 (0.76–1.32)	0.970	0.91 (0.52–1.60)	0.750
Hepatic insufficiency	72 (33.8)	1.25 (0.94–1.67)	0.124	0.97 (0.57–1.65)	0.910
AIH	75 (35.2)	0.93 (0.70–1.24)	0.621	0.70 (0.41–1.21)	0.199
Cholestasis	48 (22.5)	1.24 (0.90–1.72)	0.193	0.87 (0.51–1.49)	0.614
PBC	56 (26.3)	0.99 (0.72–1.34)	0.921	0.62 (0.34–1.13)	0.118
Viral hepatitis	31 (14.6)	1.12 (0.76–1.64)	0.572	1.04 (0.45–2.44)	0.926
Cholelithiasis	30 (14.1)	0.94 (0.64–1.38)	0.754	0.72 (0.38–1.36)	0.311
Fatty liver	13 (6.1)	1.89 (1.07–3.33)	0.028	1.44 (0.67–3.11)	0.348
DILI	10 (4.7)	1.27 (0.67–2.40)	0.458	1.13 (0.50–2.54)	0.767
Jaundice	8 (3.8)	1.95 (0.96–3.99)	0.067	0.97 (0.40–2.34)	0.950
Hepatoma	14 (6.6)	0.71 (0.41–1.22)	0.209	0.61 (0.25–1.48)	0.271
Number of disease			0.336		0.270
2 (vs. 1)	58 (27.2)	1.17 (0.83–1.66)	0.373	1.67 (0.89–3.13)	0.110
>2 (vs. 1)	78 (36.6)	1.27 (0.92–1.76)	0.144	2.14 (0.75–6.16)	0.156
Comorbidities, n (%)					
Hypertension	34 (16.0)	0.61 (0.42–0.89)	0.010	0.65 (0.31–1.33)	0.235
Diabetes	54 (25.4)	1.21 (0.89–1.65)	0.222	1.40 (0.77–2.54)	0.274
Osteoporosis	26 (12.2)	0.84 (0.56–1.27)	0.401	0.88 (0.45–1.69)	0.692
AID	24 (11.3)	0.90 (0.59–1.38)	0.624	0.94 (0.43–2.08)	0.887
Hyperlipidemia	13 (6.1)	0.78 (0.44–1.37)	0.385	0.91 (0.40–2.07)	0.828
Pneumonia	11 (5.2)	0.68 (0.37–1.27)	0.226	1.29 (0.47–3.60)	0.622
CAD	29 (13.6)	0.96 (0.65–1.42)	0.825	1.22 (0.68–2.20)	0.499
Cancer	9 (4.2)	0.90 (0.46–1.76)	0.756	0.86 (0.34–2.17)	0.747
Number of comorbidity			0.240		0.943
1 (vs. 0)	67 (31.5)	0.99 (0.72–1.35)	0.939	0.95 (0.53–1.69)	0.856
>1 (vs. 0)	49 (23.0)	0.75 (0.53–1.07)	0.110	0.82 (0.25–2.72)	0.744
Drug combinations, n (%)					
Antiviral	27 (12.7)	1.08 (0.72–1.62)	0.704	0.66 (0.18–2.51)	0.545
Glucocorticoid	26 (12.2)	0.98 (0.65–1.49)	0.941	0.93 (0.24–3.66)	0.914
Immunosuppressant	17 (8.0)	0.88 (0.53–1.44)	0.604	1.03 (0.31–3.40)	0.961
Statin	10 (4.7)	0.67 (0.36–1.28)	0.225	0.76 (0.17–3.30)	0.712
Spironolactone	10 (4.7)	1.09 (0.58–2.07)	0.787	0.97 (0.27–3.46)	0.961
CCB	9 (4.2)	0.88 (0.45–1.71)	0.698	1.97 (0.45–8.74)	0.371
ARB	8 (3.8)	0.49 (0.24–1.00)	0.051	0.63 (0.16–2.39)	0.492
Prophylactic drug	6 (2.8)	0.67 (0.30–1.52)	0.342	0.76 (0.20–2.87)	0.684
Number of drug			0.230		0.326
1 (vs. 0)	52 (24.4)	1.10 (0.80–1.52)	0.561	1.53 (0.48–4.91)	0.475
>1 (vs. 0)	28 (13.1)	0.74 (0.49–1.11)	0.148	1.16 (0.10–13.66)	0.906

Values are median (IQR) or number (percentage).HR, hazard ratio; CI, Confidence Interval. *p* values were calculated by Cox proportional hazards model. Bold values signifies *p* < 0.05. UDCA, ursodeoxycholic acid; BMI, body mass index; AIH, autoimmune hepatitis; PBC, primary biliary cirrhosis; DILI, drug induced liver injury; CAD, coronary artery disease; AID, autoimmune disease; CCB, calcium channel blockers; ARB, angiotensin receptor blocker.

## Discussion

### Chemopreventive effectiveness of UDCA

This retrospective study analyzed 1,040 outpatients prescribed with UDCA, marking the first investigation into UDCA’s association with the development of COVID-19, including infection, symptoms, severity, and prognosis. Our results indicate that UDCA use is associated with a significantly lower incidence of SARS-CoV-2 infection in both the full UDCA-exposure group and a matched UDCA-exposure group, compared to a control group with UDCA non-exposure. Additionally, UDCA mitigated the impact of COVID-19 by alleviating symptoms, reducing severity, and shortening recovery time. This suggests a protective effect of UDCA in outpatients, potentially reducing SARS-CoV-2 infection and improving COVID-19 outcomes.

Our findings complement previous studies that have illuminated the potential role of UDCA-inhibited FXR in improving COVID-19-related outcomes, both in animal models and specific human cohorts. A small cohort study comprising 31 participants with cholestatic liver disease taking UDCA reported reduced hospitalization rates, ICU admissions, and mortality compared to 155 propensity score (PS)-matched controls (Brevini et al., 2023). Another validation study in 24 liver transplant recipients found that UDCA exposure was associated with a significant decrease in moderate to severe cases of COVID-19 (Brevini et al., 2023). Furthermore, an additional study involving 1,607 UDCA-exposed participants and 1,607 controls with cirrhosis showed that UDCA exposure was linked to a reduction in both the development of SARS-CoV-2 infection and the severity of symptomatic COVID-19, including moderate, severe, and critical cases (John et al., 2023b). In summary, our research preliminarily shows the chemopreventive effectiveness of UDCA against SARS-CoV-2 infection into the outpatient cohort.

### Risk factors for COVID-19 with UDCA exposure

Our investigation identified age over 60 as a significant risk factor for increased SARS-CoV-2 infection in UDCA users. This observation aligns with most existing studies that do not specifically focus on UDCA exposure. Previous research has consistently shown that the risk of COVID-19, including the number of cases and severity, increases with age (Bai et al., 2021), a pattern also observed during the 2003 SARS epidemic (Anderson et al., 2004). In a study in 146 capital cities, higher *per capita* clinical cases were projected in cities with older populations, compared to a higher prevalence of subclinical infections in cities with younger demographics (Davies et al., 2020).

In particular, our research suggests that age stratification does not significantly affect the recovery duration from COVID-19. A systematic review that included 70 primary studies from more than 400,000 participants revealed a linear increase in the age-related risk of COVID-19 hospital mortality, case mortality, and hospitalization rates of 5.7%, 7.4%, and 3.4% per year, respectively (Romero et al., 2021). In contrast, another study involving 10,551 COVID-19 hospitalizations indicated a minimal contribution of age to critical illness outcomes (Valero-Bover et al., 2023). Similarly, a meta-analysis including 18 articles with 819,884 COVID-19 survivors did not support an association between advancing age and COVID-19 severity (Notarte et al., 2022). Given these equivocal findings, more research is necessary with larger sample sizes, well-designed stratification, and long-term studies focusing on UDCA exposure.

We observed that smokers among UDCA users not only exhibited a lower infection rate but also experienced a significantly shorter recovery duration from COVID-19. Although extensive evidence suggests that smoking may increase the risk of respiratory tract infections (van Zyl-Smit et al., 2010a), the specific impact of tobacco on SARS-CoV-2 infection and disease progression remains ambiguous. For example, a comprehensive UK study identified a higher risk of death in smokers compared to non-smokers (The OpenSAFELY Collaborative et al., 2020). Mechanistic studies have hypothesized that this increased susceptibility could be due to an upregulation of the ACE2 receptor, which facilitates the entry of SARS-CoV-2 into the host mucosa, leading to active infection (Leung et al., 2020). In contrast, a case-control study assessing the clinical outcomes of COVID-19 in smokers found that smoking decreased the risk of symptomatic infection (Saurabh et al., 2021). Another meta-analysis reported that the prevalence of current smoking among hospitalized COVID-19 patients in China was significantly lower than in the general population (Farsalinos et al., 2020). Potential protective mechanisms were proposed, including cross-protection from frequent upper respiratory tract infections common among smokers or the immunomodulatory effects of nicotine.

Additionally, a meta-analysis indicated that while the rate of SARS-CoV-2 infection was lower in smokers, the severity of hospitalization, disease severity, and mortality were higher (Simons et al., 2021). A review did not identify smoking as a risk factor for infection but found that it was associated with an increased risk of severe disease requiring mechanical ventilation or resulting in death (Vardavas and Nikitara, 2020). Another meta-analysis found no significant association between current smoking and disease severity (Lippi and Henry, 2020). Therefore, while our findings suggest that smoking may be associated with lower infection rates and shorter recovery periods in COVID-19 patients with UDCA exposure, further research with larger sample sizes and in-depth mechanistic studies is needed for confirmation.

In our study, patients who received UDCA for less than 1 month exhibited a significantly higher rate of COVID-19 infection compared to those treated for 1 month or more. However, this duration of UDCA administration did not considerably influence COVID-19 recovery time. Currently, limited studies address the correlation between the duration of UDCA treatment and COVID-19 outcomes. A small study involving eight healthy volunteers showed that a standard daily dose of UDCA at 15 mg/kg for 5 days reduced ACE2 levels in the nasal epithelium (Brevini et al., 2023). Still, this finding is insufficient to conclude the duration of UDCA treatment for the protective efficacy against COVID-19. At least 1 month of regular UDCA administration may be required to combat SARS-CoV-2 infection effectively, while a longer treatment duration is necessary to affect recovery positively.

Our study found that compliance with UDCA dosage did not significantly affect infection rates or recovery from COVID-19. Estimated daily doses for patients not strictly adhering to prescriptions ranged from 5 to 20 mg/kg. A larger study demonstrated that a 5 mg/kg increase in UDCA dosage was correlated with a reduction in SARS-CoV-2 infection and the severity of COVID-19 symptoms (John et al., 2023b). Therefore, a minimum daily dose of 5 mg/kg UDCA might lead to the observed insignificant effect of compliance.

Our results indicated that diabetes significantly increased susceptibility to SARS-CoV-2, even with UDCA exposure. Numerous studies reported that patients with type 2 diabetes are more prone to SARS-CoV-2 infection, with higher severity and mortality rates compared to non-diabetic individuals (Cariou et al., 2020; Shi et al., 2020). Moreover, COVID-19 patients with diabetes may face increased risks of acute metabolic complications (Kamrath et al., 2020), and require higher insulin doses (Guo W. et al., 2020). Potential mechanisms include elevated human ACE2 in type 2 diabetes patients (Wu et al., 2021), coupled with reduced insulin secretion and induced pancreatic β cell apoptosis due to SARS-CoV-2 infection (Muller et al., 2021). These factors lead to low-grade chronic inflammation and impaired immune function (Aluganti and Singla, 2022; Pelle et al., 2022), increasing the risk and severity of infection in diabetic individuals (Apicella et al., 2020; Singh et al., 2020). Although diabetes is a significant risk factor for COVID-19 infection in our UDCA-exposed population, it did not significantly affect recovery from the disease. This observation is consistent with a previous study that found no difference in COVID-19 severity or hospital stay duration based on diabetes status (Bajpeyi et al., 2022).

Our study found that participants with preexisting conditions such as CAD and hypertension exhibited higher SARS-CoV-2 infection rates and longer COVID-19 recovery times. The link between COVID-19 and cardiovascular disease (CVD) is well documented, with patients having preexisting CVD facing more severe complications and higher mortality rates (Nishiga et al., 2020). Furthermore, COVID-19 may exacerbate the development of CVD (Guo T. et al., 2020). CAD and hypertension, as common forms of CVD, are associated with increased morbidity and mortality in COVID-19 patients (Zhu et al., 2020; Ko et al., 2021; Al-Qudimat et al., 2023; Dagan et al., 2023).

The introduction of various COVID-19 vaccines has been successful in preventing symptomatic infection, severe symptoms, hospitalization, and COVID-19-related deaths (Sobczak and Pawliczak, 2022). Interestingly, our study did not find a significant correlation between vaccination and SARS-CoV-2 infection in UDCA-exposed outpatients, even among those who received complete or booster vaccinations. Additionally, in univariate analysis, a substantial reduction in COVID-19 recovery time was observed only in patients who received complete or booster vaccination but not in multivariate analysis. This suggests that UDCA might substitute or overlap the well-established preventive efficacy of COVID-19 vaccines. This is consistent with previous literature indicating similar associations between UDCA exposure and COVID-19-related outcomes among fully vaccinated and unvaccinated participants (John et al., 2023b).

Preexisting chronic liver disease was associated with a poorer prognosis in COVID-19 patients (Ji et al., 2020; Kim et al., 2022). This could be attributed to inflammatory liver disease increasing the risk of a “cytokine storm” (Jagirdhar et al., 2023). However, our findings indicated that infection and recovery from COVID-19 in patients with liver diseases, except those with fatty liver disease, were not significantly different from non-afflicted patients, even with UDCA exposure, except for the shorter recovery time in individuals with fatty liver disease in univariate analysis. This might be because outpatients generally have milder liver diseases, insufficient to impact COVID-19 progression. Additionally, considering UDCA’s protective effects on hepatocytes, including reducing cholestasis, improving liver function, and alleviating hepatic fibrosis (Ye et al., 2020), its preventive effects on both liver diseases and COVID-19 could counteract the negative impact of liver diseases on COVID-19.

### The impact of UDCA on COVID-19 symptoms

A comprehensive study documented 32 symptoms among 4,990 individuals who tested positive for COVID-19 during the predominance of the Omicron variant. The most commonly reported symptoms included runny nose (76.5%), headache (74.7%), sore throat (70.5%), sneezing (63.0%), cough (49.8%), and hoarse voice (42.6%) (Menni et al., 2022). In our study, the primary symptoms observed among UDCA users aligned with this report, including runny nose, sore throat, cough, fever, and others. Consequently, we hypothesize that UDCA has a negligible impact on the type of clinical symptoms exhibited by patients with COVID-19.

In October 2021, the WHO defined the post-COVID-19 condition as symptoms persisting for 3 months after infection, lasting at least 2 months, and are not attributable to an alternative diagnosis. A preprint study revealed that in 2020 and 2021, approximately 144.7 million people globally (95% uncertainty interval [UI]: 54.8–312.9 million), corresponding to 3.7% (UI: 1.4–8.0) of all infections, suffered from fatigue (51.0%, UI: 16.9–92.4), respiratory (60.4%, UI: 18.9–89.1), and cognitive (35.4%, UI: 9.4–75.1) symptoms associated with long COVID-19. The pathophysiology of long COVID-19 is believed to involve a prolonged low-grade infection state, a hyperimmune response, coagulation/vasculopathy, endocrine and autonomic dysregulation, and maladaptation of the ACE-2 pathway (Nalbandian et al., 2021). In our study, the median time from the onset of SARS-CoV-2 infection to the follow-up was 65.0 days (range: 60.0–75.0 days). Among our participants, 9.8% (74 of 755 individuals) reported ongoing COVID-19-related symptoms, including fatigue, cough, hypoesthesia, muscle/joint pain, dizziness, palpitations, dyspnea, expectoration, sore throat, hyposmia, and hypogeusia. This observation further suggests that UDCA may not significantly influence the range of COVID-19-related symptoms experienced by patients.

### Strengths

This study is among the first to explore the association between UDCA use and COVID-19 development, including infection, symptoms, severity, prognosis, and risk factors and symptoms in outpatients with UDCA exposure. As COVID-19 symptoms tend to be milder in vaccinated and unvaccinated patients, outpatients, as opposed to inpatients, are likely to represent the majority of future COVID-19 cases. The information gained from outpatient populations is crucial for improving public health protection and mitigating the impact of future pandemics. Furthermore, our study uniquely selected outpatients prescribed UDCA who did not take the medication as a control group rather than those without a UDCA prescription. This approach aimed to minimize case selection bias, considering that both groups had similar indications for UDCA use. To further reduce confounding, we used a PSM analysis using the nearest neighbor matching algorithm, which yielded well-matched groups.

### Limitations

Our study, a retrospective cohort analysis, has inherent limitations. First, the retrospective nature poses challenges, such as residual confounding. UDCA was prescribed to patients in an outpatient setting, not primarily for COVID-19. Data collection relied heavily on self-reports, and despite cross-referencing with medical records, memory bias and unmeasured confounding factors could only partially be eliminated. Second, the study’s small patient cohort and the single-center execution limit the generalizability of our findings. The efficacy and safety of UDCA for COVID-19 warrant further investigation in large-scale multicenter studies. However, our data may provide valuable benchmarks for future research design. Third, the study lacked statistical power to detect differences in COVID-19-related mortality rates. Given the widespread vaccine use, reduced viral pathogenicity, and milder disease courses in the non-hospitalized population, there were no COVID-19-related deaths in our study groups. This highlights the need for more comprehensive studies to assess UDCA’s impact on severe COVID-19 outcomes.

## Conclusion

As COVID-19 becomes more endemic, the ongoing battle with SARS-CoV-2 persists. The focus is increasingly shifting towards disease prevention and control. UDCA may offer chemopreventive benefits against COVID-19 in outpatients, including reducing infection and symptom severity and shortening disease duration. Factors such as older age, insufficient duration of UDCA treatment, and comorbidities such as diabetes mellitus and CAD significantly increased the SARS-CoV-2 infection rates. In contrast, hypertension was associated with a prolonged COVID-19 recovery. Smoking decreased infection rates, and smoking, vaccination, and fatty liver disease were associated with shorter recovery periods. UDCA showed minimal impact on the variety of COVID-19-related symptoms. More extensive and longer-term clinical studies are needed to assess UDCA on COVID-19 prevention or treatment.

## Data Availability

The original contributions presented in the study are included in the article/Supplementary Material, further inquiries can be directed to the corresponding authors.

## References

[B1] Al-Qudimat A. R. Ameen A. Sabir D. M. Alkharraz H. Elaarag M. Althani A. (2023). The Association of Hypertension With Increased Mortality Rate During the COVID-19 Pandemic: An Update With Meta-Analysis. J. Epidemiol. Glob. Health. 13 (3), 495–503. 10.1007/s44197-023-00130-3 37318701 PMC10469154

[B2] Aluganti N. C. Singla D. K. (2022). Mechanisms of COVID-19 Pathogenesis in Diabetes. Am. J. Physiol.-Heart Circ. Physiol. 323 (3), H403–H420. 10.1152/ajpheart.00204.2022 35776683 PMC9359655

[B3] Anderson R. M. Fraser C. Ghani A. C. Donnelly C. A. Riley S. Ferguson N. M. (2004). Epidemiology, Transmission Dynamics and Control of SARS: The 2002-2003 Epidemic. Philos. Trans. R. Soc. B-Biol. Sci. 359 (1447), 1091–1105. 10.1098/rstb.2004.1490 PMC1693389 15306395

[B4] Apicella M. Campopiano M. C. Mantuano M. Mazoni L. Coppelli A. Del P. S. (2020). COVID-19 in People With Diabetes: Understanding the Reasons for Worse Outcomes. Lancet Diabetes Endocrinol. 8 (9), 782–792. 10.1016/S2213-8587(20)30238-2 32687793 PMC7367664

[B5] Bai Y. Gao L. Wang X. Zhong L. Li J. Ding S. (2021). Epidemiological Characteristics and Clinical Manifestations of Pediatric Patients With COVID-19 in China: A Multicenter Retrospective Study. Pediat. Invest. 5 (3), 203–210. 10.1002/ped4.12282 PMC8441896 34540320

[B6] Bajpeyi S. Mossayebi A. Kreit H. Cherukuri S. Mandania R. A. Concha J. B. (2022). Unmanaged Diabetes and Elevated Blood Glucose Are Poor Prognostic Factors in the Severity and Recovery Time in Predominantly Hispanic Hospitalized COVID-19 Patients. Front. Endocrinol. 13, 861385. 10.3389/fendo.2022.861385 PMC9309175 35898451

[B7] Brevini T. Maes M. Webb G. J. John B. V. Fuchs C. D. Buescher G. (2023). FXR Inhibition May Protect From SARS-CoV-2 Infection by Reducing ACE2. Nature 615 (7950), 134–142. 10.1038/s41586-022-05594-0 36470304 PMC9977684

[B8] Cariou B. Hadjadj S. Wargny M. Pichelin M. Al-Salameh A. Allix I. (2020). Phenotypic Characteristics and Prognosis of Inpatients with COVID-19 and Diabetes: the CORONADO Study. Diabetologia 63 (8), 1500–1515. 10.1007/s00125-020-05180-x 32472191 PMC7256180

[B9] Contini C. Rotondo J. C. Perna B. Guarino M. De Giorgio R. (2023). Special Issue: Advances in SARS-cov-2 Infection. Microorganisms 11 (4), 1048. 10.3390/microorganisms11041048 37110471 PMC10145712

[B10] Dagan M. Cheung K. Quine E. Gard E. Johnston R. Barker S. (2023). Coronary Artery Disease Risk Prediction in Patients With Severe Aortic Stenosis: Development and Validation of the Aortic Stenosis-Coronary Artery Disease (AS-CAD) Score. Am. J. Cardiol. 205, 134–140. 10.1016/j.amjcard.2023.07.168 37598598

[B11] Davies N. G. Klepac P. Liu Y. Prem K. Jit M. Eggo R. M. (2020). Age-Dependent Effects in the Transmission and Control of COVID-19 Epidemics. Nat. Med. 26 (8), 1205–1211. 10.1038/s41591-020-0962-9 32546824

[B12] Farsalinos K. Barbouni A. Niaura R. (2020). Systematic Review of the Prevalence of Current Smoking Among Hospitalized COVID-19 Patients in China: Could Nicotine be a Therapeutic Option? Intern. Emerg. Med. 15 (5), 845–852. 10.1007/s11739-020-02355-7 32385628 PMC7210099

[B13] Ferreira R. D. John B. V. (2023). Viral Vector Vaccines Are Victorious Against COVID-19 in Patients with Cirrhosis. Dig. Dis. Sci. 68 (2), 349–351. 10.1007/s10620-022-07644-z 35947303 PMC9363849

[B14] Gaziano L. Giambartolomei C. Pereira A. C. Gaulton A. Posner D. C. Swanson S. A. (2021). Actionable Druggable Genome-Wide Mendelian Randomization Identifies Repurposing Opportunities for COVID-19. Nat. Med. 27 (4), 668–676. 10.1038/s41591-021-01310-z 33837377 PMC7612986

[B15] Guo T. Fan Y. Chen M. Wu X. Zhang L. He T. (2020b). Cardiovascular Implications of Fatal Outcomes of Patients With Coronavirus Disease 2019 (COVID-19). Jama Cardiol. 5 (7), 811–818. 10.1001/jamacardio.2020.1017 32219356 PMC7101506

[B16] Guo W. Li M. Dong Y. Zhou H. Zhang Z. Tian C. (2020a). Diabetes is a Risk Factor for the Progression and Prognosis of COVID-19. Diabetes-Metab. Res. Rev. 36 (7), e3319. 10.1002/dmrr.3319 32233013 PMC7228407

[B17] Jagirdhar G. Pattnaik H. Banga A. Qasba R. K. Rama K. Reddy S. T. (2023). Association of Non-Alcoholic Fatty Liver Disease and Metabolic-Associated Fatty Liver Disease With COVID-19-Related Intensive Care Unit Outcomes: A Systematic Review and Meta-Analysis. Med. Lith. 59 (7), 1239. 10.3390/medicina59071239 PMC10386363 37512051

[B18] Ji D. Zhang D. Yang T. Mu J. Zhao P. Xu J. (2020). Effect of COVID-19 on Patients With Compensated Chronic Liver Diseases. Hepatol. Int. 14 (5), 701–710. 10.1007/s12072-020-10058-6 32734407 PMC7391917

[B19] John B. V. Bastaich D. Webb G. Brevini T. Moon A. Ferreira R. D. (2023b). Ursodeoxycholic Acid is Associated With a Reduction in SARS-CoV-2 Infection and Reduced Severity of COVID-19 in Patients With Cirrhosis. J. Intern. Med. 293 (5), 636–647. 10.1111/joim.13630 37018129 PMC12036735

[B20] John B. V. Bastaich D. R. Ferreira R. D. Doshi A. Taddei T. H. Kaplan D. E. (2023a). COVID-19 Vaccine Effectiveness and Community Prevalence of Alpha, Delta and Omicron Variants in Patients With Cirrhosis. Gut 72 (9), 1800–1802. 10.1136/gutjnl-2022-327799 36562753

[B21] John B. V. Deng Y. Scheinberg A. Mahmud N. Taddei T. H. Kaplan D. (2021). Association of BNT162b2 mRNA and mRNA-1273 Vaccines With COVID-19 Infection and Hospitalization Among Patients With Cirrhosis. Jama Intern. Med. 181 (10), 1306–1314. 10.1001/jamainternmed.2021.4325 34254978 PMC8278308

[B22] John B. V. Sidney B. A. T. Moon A. Taddei T. H. Kaplan D. E. Dahman B. (2022). Effectiveness of COVID-19 Viral Vector Ad.26.COV2. S Vaccine and Comparison With mRNA Vaccines in Cirrhosis. Clin. Gastroenterol. Hepatol. 20 (10), 2405–2408.e3. 10.1016/j.cgh.2022.05.038 35716904 PMC9212810

[B23] Kamrath C. Monkemoller K. Biester T. Rohrer T. R. Warncke K. Hammersen J. (2020). Ketoacidosis in Children and Adolescents with Newly Diagnosed Type 1 Diabetes During the COVID-19 Pandemic in Germany. Jama-J. Am. Med. Assoc. 324 (8), 801–804. 10.1001/jama.2020.13445 PMC7372511 32702751

[B24] Kim M. K. Lee B. Choi Y. Y. Um J. Lee K. S. Sung H. K. (2022). Clinical Characteristics of 40 Patients Infected With the SARS-CoV-2 Omicron Variant in Korea. J. Korean Med. Sci. 37 (3), e31. 10.3346/jkms.2022.37.e31 35040299 PMC8763884

[B25] Ko J. Y. Danielson M. L. Town M. Derado G. Greenlund K. J. Kirley P. D. (2021). Risk Factors for Coronavirus Disease 2019 (COVID-19)-Associated Hospitalization: COVID-19-Associated Hospitalization Surveillance Network and Behavioral Risk Factor Surveillance System. Clin. Infect. Dis. 72 (11), e695–e703. 10.1093/cid/ciaa1419 32945846 PMC7543371

[B26] Leung J. M. Yang C. X. Tam A. Shaipanich T. Hackett T. L. Singhera G. K. (2020). ACE-2 Expression in the Small Airway Epithelia of Smokers and COPD Patients: Implications for COVID-19. Eur. Resp. J. 55 (5), 2000688. 10.1183/13993003.00688-2020 PMC7144263 32269089

[B27] Lippi G. Henry B. M. (2020). Active Smoking is Not Associated With Severity of Coronavirus Disease 2019 (COVID-19). Eur. J. Intern. Med. 75, 107–108. 10.1016/j.ejim.2020.03.014 32192856 PMC7118593

[B28] Lippi G. Sanchis-Gomar F. Henry B. M. (2023). COVID-19 and its Long-Term Sequelae: What do We Know in 2023? Pol. Intern. Med. 133 (4), 16402. 10.20452/pamw.16402 36626183

[B29] Looi M. (2023). Covid-19: Scientists Sound Alarm Over New BA.2.86 “Pirola” Variant. Bmj-British Med. J. 382, 1964. 10.1136/bmj.p1964 37620014

[B30] Mahase E. (2023). Covid-19: New “Pirola” Variant BA.2.86 Continues to Spread in UK and US. Bmj-British Med. J. 382, 2097. 10.1136/bmj.p2097 37704230

[B31] Menni C. Valdes A. M. Polidori L. Antonelli M. Penamakuri S. Nogal A. (2022). Symptom Prevalence, Duration, and Risk of Hospital Admission in Individuals Infected With SARS-CoV-2 During Periods of Omicron and Delta Variant Dominance: A Prospective Observational Study from the ZOE COVID Study. Lancet 399 (10335), 1618–1624. 10.1016/S0140-6736(22)00327-0 35397851 PMC8989396

[B32] Muller J. A. Gross R. Conzelmann C. Kruger J. Merle U. Steinhart J. (2021). SARS-CoV-2 Infects and Replicates in Cells of the Human Endocrine and Exocrine Pancreas. Nat. Metab. 3 (2), 149–165. 10.1038/s42255-021-00347-1 33536639

[B33] Nalbandian A. Sehgal K. Gupta A. Madhavan M. V. McGroder C. Stevens J. S. (2021). Post-Acute COVID-19 Syndrome. Nat. Med. 27 (4), 601–615. 10.1038/s41591-021-01283-z 33753937 PMC8893149

[B34] Nishiga M. Wang D. W. Han Y. Lewis D. B. Wu J. C. (2020). COVID-19 and Cardiovascular Disease: From Basic Mechanisms to Clinical Perspectives. Nat. Rev. Cardiol. 17 (9), 543–558. 10.1038/s41569-020-0413-9 32690910 PMC7370876

[B35] Notarte K. I. de Oliveira M. Peligro P. J. Velasco J. V. Macaranas I. Ver A. T. (2022). Age, Sex and Previous Comorbidities as Risk Factors Not Associated with SARS-CoV-2 Infection for Long COVID-19: A Systematic Review and Meta-Analysis. J. Clin. Med. 11 (24), 7314. 10.3390/jcm11247314 36555931 PMC9787827

[B36] Pagani I. Ghezzi S. Alberti S. Poli G. Vicenzi E. (2023). Origin and Evolution of SARS-cov-2. Eur. Phys. J. Plus 138 (2), 157. 10.1140/epjp/s13360-023-03719-6 36811098 PMC9933829

[B37] Pelle M. C. Zaffina I. Provenzano M. Moirano G. Arturi F. (2022). COVID-19 and Diabetes-Two Giants Colliding: From Pathophysiology to Management. Front. Endocrinol. 13, 974540. 10.3389/fendo.2022.974540 PMC9437522 36060943

[B38] Romero S. K. Reissig D. Petereit-Haack G. Schmauder S. Nienhaus A. Seidler A. (2021). The Isolated Effect of Age on the Risk of COVID-19 Severe Outcomes: A Systematic Review With Meta-Analysis. Bmj Glob. Health 6 (12), e006434. 10.1136/bmjgh-2021-006434 PMC8678541 34916273

[B39] Saurabh S. Verma M. K. Gautam V. Kumar N. Jain V. Goel A. D. (2021). Tobacco, Alcohol Use and Other Risk Factors For Developing Symptomatic COVID-19 vs Asymptomatic SARS-CoV-2 Infection: A Case-Control Study From Western Rajasthan, India. Trans. Roy. Soc. Trop. Med. Hyg. 115 (7), 820–831. 10.1093/trstmh/traa172 33444432 PMC7928693

[B40] Shi Q. Zhang X. Jiang F. Zhang X. Hu N. Bimu C. (2020). Clinical Characteristics and Risk Factors for Mortality of COVID-19 Patients with Diabetes in Wuhan, China: A Two-Center, Retrospective Study. Diabetes Care 43 (7), 1382–1391. 10.2337/dc20-0598 32409504

[B41] Simons D. Shahab L. Brown J. Perski O. (2021). The Association of Smoking Status With SARS-CoV-2 Infection, Hospitalization and Mortality From COVID-19: A Living Rapid Evidence Review With Bayesian Meta-Analyses (Version 7). Addiction 116 (6), 1319–1368. 10.1111/add.15276 33007104 PMC7590402

[B42] Singh A. K. Gupta R. Ghosh A. Misra A. (2020). Diabetes in COVID-19: Prevalence, Pathophysiology, Prognosis and Practical Considerations. Diabetes Metab. Syndr-Clin. Res. Rev. 14 (4), 303–310. 10.1016/j.dsx.2020.04.004 PMC7195120 32298981

[B43] Sobczak M. Pawliczak R. (2022). COVID-19 Vaccination Efficacy in Numbers Including SARS-CoV-2 Variants and Age Comparison: A Meta-Analysis of Randomized Clinical Trials. Ann. Clin. Microbiol. Antimicrob. 21 (1), 32. 10.1186/s12941-022-00525-3 35786399 PMC9250750

[B44] The OpenSAFELY Collaborative, Elizabeth W. Alex J. W. Krishnan B. Seb B. Chris B. (2020). OpenSAFELY: Factors Associated With COVID-19-Related Hospital Death in the Linked Electronic Health Records of 17 Million Adult NHS Patients. Med. 2020, 2025. 10.1101/2020.05.06.20092999

[B45] Valero-Bover D. Monterde D. Carot-Sans G. Cainzos-Achirica M. Comin-Colet J. Vela E. (2023). Is Age the Most Important Risk Factor in COVID-19 Patients? The Relevance of Comorbidity Burden: A Retrospective Analysis of 10,551 Hospitalizations. Clin. Epidemiol. 15, 811–825. 10.2147/CLEP.S408510 37408865 PMC10319286

[B46] van Zyl-Smit R. N. Brunet L. Pai M. Yew W. W. (2010a). The Convergence of the Global Smoking, COPD, Tuberculosis, HIV, and Respiratory Infection Epidemics. Infect. Dis. Clin. North Am. 24 (3), 693–703. 10.1016/j.idc.2010.04.012 20674799 PMC2914695

[B47] Vardavas C. I. Nikitara K. (2020). COVID-19 and Smoking: A Systematic Review of the Evidence. Tob. Induc. Dis. 18, 20. 10.18332/tid/119324 32206052 PMC7083240

[B48] Wang Q. Guo Y. Iketani S. Nair M. S. Li Z. Mohri H. (2022). Antibody Evasion by SARS-CoV-2 Omicron Subvariants BA.2.12.1, BA.4 and BA.5. Nature 608 (7923), 603–608. 10.1038/s41586-022-05053-w 35790190 PMC9385487

[B49] Wong G. L. Hui V. W. Yip T. C. Lui G. C. Hui D. S. Wong V. W. (2023). Minimal Risk of Drug-Induced Liver Injury With Molnupiravir and Ritonavir-Boosted Nirmatrelvir. Gastroenterology 164 (1), 151–153. 10.1053/j.gastro.2022.09.008 36126688 PMC9568277

[B50] Wu C. T. Lidsky P. V. Xiao Y. Lee I. T. Cheng R. Nakayama T. (2021). SARS-CoV-2 Infects Human Pancreatic β Cells and Elicits β Cell Impairment. Cell. Metab. 33 (8), 1565–1576.e5. 10.1016/j.cmet.2021.05.013 34081912 PMC8130512

[B51] Ye H. L. Zhang J. W. Chen X. Z. Wu P. B. Chen L. Zhang G. (2020). Ursodeoxycholic Acid Alleviates Experimental Liver Fibrosis Involving Inhibition of Autophagy. Life Sci. 242, 117175. 10.1016/j.lfs.2019.117175 31843528

[B52] Zampino R. Mele F. Florio L. L. Bertolino L. Andini R. Galdo M. (2020). Liver Injury in Remdesivir-Treated COVID-19 Patients. Hepatol. Int. 14 (5), 881–883. 10.1007/s12072-020-10077-3 32725454 PMC7386240

[B53] Zhu N. Zhang D. Wang W. Li X. Yang B. Song J. (2020). A Novel Coronavirus From Patients With Pneumonia in China, 2019. N. Engl. J. Med. 382 (8), 727–733. 10.1056/NEJMoa2001017 31978945 PMC7092803

