# Supplementary material for: Evaluating the protective effectiveness and risk factors of ursodeoxycholic acid on COVID-19 among outpatients
Source: Front Pharmacol. 2024 Jul 31;15:1381830. doi: 10.3389/fphar.2024.1381830 (PMC11321974; doi:10.3389/fphar.2024.1381830)
Supplement: Supplementary file 1 [file DataSheet1.docx]

Supplement 1.

Patient ID NO.__________ **Questionnaire of Ursodeoxycholic Acid (UDCA) for the Prevention and Treatment of COVID-19 in Outpatients- Branch 1 (Patient Information)**

1. Full Name__________
2. Date__________
3. Research Personnel_________
4. Whether the patient died? ☐Yes (End the survey) ☐No
5. Is the patient willing to participate in this survey? ☐Yes ☐No (End the survey)
6. Did you take UDCA? ☐Yes ☐No
7. Did you take UDCA as the doctor's prescription?

☐Yes_________________ (Start date, Stop date)

☐No__________________ (Dosage and method of administration, Start date, Stop date)

1. Were you infected with COVID-19 (Mainly refers to the infection situation in November to December 2022)?

☐Yes ___________________ (Infection date)

→Method of diagnosis: ☐RT-qPCR ☐RAT ☐Both (go to Branch 2)

☐No

→Method of diagnosis: ☐RT-qPCR ☐RAT ☐Both (go to Branch 3)

Patient ID NO.__________
**Questionnaire of Ursodeoxycholic Acid (UDCA) for the Prevention and Treatment of COVID-19 in Outpatients - Branch 2 (Infected Patients)**

1. Did your RT-QPCR/RAT test turn negative?

☐Yes__________________ (Date)

→Method of diagnosis: ☐RT-qPCR ☐RAT ☐Both

☐No

→Method of diagnosis: ☐RT-qPCR ☐RAT ☐Both

☐Not tested

1. Did you have symptoms during infection?

☐Yes________________ (Onset date) (go to question 3)

☐No (go to question 4)

1. Clinical manifestations and severity

| **Mandatory questions** | | |
| --- | --- | --- |
| **Symptom** | **Status** | |
| Fever | ☐Yes  Severity→☐1 ☐2 ☐3 ☐4 ☐5 ☐6 ☐7 ☐8 ☐9 ☐10  (Duration <days>) | ☐No |
| Cough | ☐Yes  Severity→☐1 ☐2 ☐3 ☐4 ☐5 ☐6 ☐7 ☐8 ☐9 ☐10  (Duration <days>) | ☐No |
| Headache | ☐Yes  Severity→☐1 ☐2 ☐3 ☐4 ☐5 ☐6 ☐7 ☐8 ☐9 ☐10  (Duration <days>) | ☐No |
| Fatigue | ☐Yes  Severity→☐1 ☐2 ☐3 ☐4 ☐5 ☐6 ☐7 ☐8 ☐9 ☐10  (Duration <days>) | ☐No |
| Expectoration | ☐Yes  Severity→☐1 ☐2 ☐3 ☐4 ☐5 ☐6 ☐7 ☐8 ☐9 ☐10  (Duration <days>) | ☐No |
| Sore throat | ☐Yes  Severity→☐1 ☐2 ☐3 ☐4 ☐5 ☐6 ☐7 ☐8 ☐9 ☐10  (Duration <days>) | ☐No |

| **Mandatory questions** | | |
| --- | --- | --- |
| **Symptom** | **Status** | |
| Muscle/joint pain | ☐Yes  Severity→☐1 ☐2 ☐3 ☐4 ☐5 ☐6 ☐7 ☐8 ☐9 ☐10  (Duration <days>) | ☐No |
| Runny nose | ☐Yes  Severity→☐1 ☐2 ☐3 ☐4 ☐5 ☐6 ☐7 ☐8 ☐9 ☐10  (Duration <days>) | ☐No |
| Anorexia | ☐Yes  Severity→☐1 ☐2 ☐3 ☐4 ☐5 ☐6 ☐7 ☐8 ☐9 ☐10  (Duration <days>) | ☐No |
| Hyposthenia | ☐Yes  Severity→☐1 ☐2 ☐3 ☐4 ☐5 ☐6 ☐7 ☐8 ☐9 ☐10  (Duration <days>) | ☐No |
| Other |  | ☐No |

1. Were you vaccinated?

☐Yes

| Type of Vaccine | | Inactivated vaccine | | | Recombinant protein subunit vaccine | adenovirus vector vaccine | | Unknown type |
| --- | --- | --- | --- | --- | --- | --- | --- | --- |
| Vaccine brands | | CoronaVac | BBIBP-CorV | WIBP-CorV | Zifivax | Convidecia | |  |
|  |  |  |  |  |  | Injection | Inhalation |  |
| First dose | | □ | □ | □ | □ | □ | **/** | □ |
| Second dose | | □ | □ | □ | □ | / | / | □ |
| Third dose | | / | / | / | □ | / | / | □ |
| booster dose | 1 | □ | □ | □ | □ | □ | □ | □ |
|  | 2 | □ | □ | □ | □ | □ | □ | □ |
| Last dose date | |  | | | | | | |

☐No

1. Did you take any prophylactic drugs before infection?

☐Yes (e.g., Thymalfasin, Thymopentin, Human Immunoglobulin (pH4))

__________________ (Dosage and duration)

☐No

1. Do you have any discomfort or symptoms currently ?

☐Yes

→Type and duration of symptoms

| □Chest distress ( Duration <days>) | | □Dyspnea |
| --- | --- | --- |
| □Respiratory pain | □Palpitation | □Muscle/Joint pain |
| □Throat discomfort | □ Sneezing | □ Runny nose |
| □ Dry cough | □ Wet cough | □ Fever |
| □Alternating Chills and Fever | | □ Limb Heaviness |
| □Hypogeusia | □Hyposmia | □Hypomnesia |
| □Headache | □Dizziness | □ Stomach pain |
| □ Nausea | □Back pain | □ Eye itchiness |
| □Diarrhea | □Anorexia | □Hyposthenia |
| □Slow response | □ Anxiety | □ Sleep Disturbance |
| □Other | | |

☐No

Patient ID NO.__________
**Questionnaire of Ursodeoxycholic Acid (UDCA) for the Prevention and Treatment of COVID-19 in Outpatients - Branch 3 (Non-infected patients)**

1. Were you vaccinated?

☐Yes

| Type of Vaccine | | Inactivated vaccine | | | Recombinant protein subunit vaccine | adenovirus vector vaccine | | Unknown type |
| --- | --- | --- | --- | --- | --- | --- | --- | --- |
| Vaccine brands | | CoronaVac | BBIBP-CorV | WIBP-CorV | Zifivax | Convidecia | |  |
|  |  |  |  |  |  | Injecti0n | Inhalation |  |
| First dose | | □ | □ | □ | □ | □ | **/** | □ |
| Second dose | | □ | □ | □ | □ | / | / | □ |
| Third dose | | / | / | / | □ | / | / | □ |
| booster dose | 1 | □ | □ | □ | □ | □ | □ | □ |
|  | 2 | □ | □ | □ | □ | □ | □ | □ |
| Last dose date | |  | | | | | | |

☐No

1. Did you take any prophylactic drugs before infection?

☐Yes (e.g., Thymalfasin, Thymopentin, Human Immunoglobulin (pH4))

__________________ (Dosage and duration)

☐No

Supplement 2. 2019-nCoV vaccine types, brands and administration regimen

| Type of Vaccine | Inactivated vaccine | | | Recombinant protein subunit vaccine | Adenovirus vector vaccine | |
| --- | --- | --- | --- | --- | --- | --- |
| Vaccine brands | CoronaVac | BBIBP-CorV | WIBP-CorV | Zifivax | Convidecia | |
| Administration regimen | Injection | | | Injection | Injection | Inhalation |
| Complete vaccination | 2 | | | 3 | 1 | |
